# The Semi-Annual Meeting of the Medical Society of Erie County

**Published:** 1858-07

**Authors:** 


*The Semi-annual Meeting of the Medical Society of Erie County,* was held in this city, at the Rooms of the Buffalo Medical Association, on Tuesday the 8th of June. The attendance was large, a goodly number of members from the country being present.

The society was called to order about 10½, A. M., by the president, Prof. Flint. The session was shorter than usual, no matters of any great importance coming before the meeting to demand its attention, and the *oration,* which is, or should constitute one of the prominent features of these gatherings, failed of delivery.

Drs. J. Fletcher Stevens, Augustus Jansen, Jessie I. Richards, William H. Butler, N. S. Lockwood, Charles Storck, Andrew C. Morey, Bernard Monahan, made applications for membership with the society, and were severally elected to membership, after a proper examination of their credentials, upon the condition that they comply with all the by-laws of the society, and the laws of the State of New York.

The committee appointed June 10th, 1856, in reference to the organization of a society for the relief of widows and orphans of medical men, *reported,* That within a few weeks past such a society had been organized under the name and title of *The Buffalo Physicians' Charitable Fund Association.*

Dr. C. C. F. Gay was appointed *orator* for the January meeting, and Dr. A. Flint, Jr., his substitute.

During the session a peremptory mandamus from the Supreme Court of the State of New York, was served upon the president, commanding the society to reinstate Dr. E. P. Gray in all the rights and privileges of a member of the society; accordingly, in compliance with the aforesaid mandamus, Dr. Gray was so reinstated.

On motion, the law papers connected with the aforesaid writ, were referred to the officers of the society for any legal advice or action which might be necessary to perfect the same, or which the case demanded.

A dinner committee, consisting of Drs. Wilcox, Gay and Hutchins, was appointed to provide a dinner for the annual session.

We have noted, we believe, all the matters of interest. The society adjourned about 1 o'clock, P. M.

We hope we shall have a session of more general interest at the next regular meeting. It is to be regretted that the scientific character of these meetings are so much lost sight of, and the business of the society be permitted to degenerate into only the legislative action necessary to protect the legal standing of the profession, by drawing the lines of distinction between regular and irregular practitioners. N.

